# Accelerated corneal cross-linking with hypo-osmolar riboflavin in thin keratoconic corneas: 2-year follow-up

**DOI:** 10.5935/0004-2749.20200049

**Published:** 2020

**Authors:** Dilay Ozek, Emine Esra Karaca, Ozlem Evren Kemer

**Affiliations:** 1 Professor, Department of Ophthalmology, Ankara Numune Education and Research Hospital, Ankara, Turkey

**Keywords:** Keratoconus, Corneal pachymetry, Cross-linking reagents, Riboflavin/therapeutic use, Ceratocone, Paquimetria corneana, Reagentes para ligações cruzadas, Riboblavina/uso terapêutico

## Abstract

**Purpose:**

This study was performed to evaluate the outcomes of accelerated corneal cross-linking in keratoconic corneas with thinnest pachymetry values of <400 µm.

**Methods:**

The study included 28 eyes of 24 patients. The uncorrected and best-corrected visual acuities (logMAR), flattest and steepest keratometric readings, central corneal thickness at the thinnest point, corneal higher-order aberrations, and contrast sensitivity were assessed before and at 1, 3, 6, 12, and 24 months after corneal cross-linking.

**Result:**

The mean best-corrected visual acuity and contrast sensitivity increased (p=0.02, p=0.03, respectively), whereas the mean uncorrected visual acuity did not significantly differ (p>0.05) at 24 months after corneal cross-linking, compared with measurements before corneal cross-linking. Although the mean flattest keratometric reading showed no significant change (p=0.58), the mean steepest keratometric reading was reduced when compared with its value before corneal cross-linking (p=0.001). No change was observed in the mean central corneal thickness at the thinnest point at 24 months after corneal cross-linking, compared with its value before corneal cross-linking (p=0.12).

**Conclusion:**

Accelerated corneal cross-linking in keratoconic eyes with thin corneas could halt the progression of keratoconus in corneas thinner than 400 µm at 24 months after treatment.

## Abstract (pt)

**Objetivo:**

Este estudo foi realizado para avaliar os resultados do *cross-linking* corneano acelerado em córneas ceratocônicas com os valores mais baixos de paquimetria <400 µm.

**Métodos:**

O estudo incluiu 28 olhos de 24 pacientes. As acuidades visuais não corrigidas e melhor corrigidas (logMAR), leituras ceratométricas mais planas e íngremes, espessura corneana central no ponto mais fino, aberrações corneanas de mais alta ordem e a sensibilidade ao contraste foram avaliadas antes e em 1, 3, 6, 12 e 24 meses após a realização do do *cross-linking*.

**Resultados:**

A média da acuidade visual melhor corrigida e a sensibilidade ao contraste aumentaram (p=0,02, p=0,03, respectivamente), enquanto a média da acuidade visual não corrigida não diferiu significativamente (p>0,05) aos 24 meses após o *cross-linking,* comparada com medidas antes do procedimento. Embora a leitura da média da ceratometria mais plana não tenha apresentado alteração significativa (p=0,58), a leitura ceratométrica mais íngreme diminuiu quando comparada ao seu valor antes do *cross-linking* (p=0,001). Não foi observada alteração na média da espessura corneana central no ponto mais fino aos 24 meses após o *cross-linking* em comparação com seu valor antes do procedimento (p=0,12).

**Conclusão:**

O *cross-linking* corneano acelerado nos olhos ceratocônicos com córneas finas pode interromper a progressão do ceratocone nas córneas mais finas que 400 µm 24 meses após o tratamento.

## INTRODUCTION

The primary aim of corneal cross-linking (CXL) is to stop the progression of corneal ectasia. Riboflavin acts as a photosensitizer in the photopolymerization process; combined with ultraviolet A (UVA) irradiation, riboflavin treatment increases the formation of intrafibrillary and interfibrillary carbonyl-based collagen covalent bonds through a molecular process that is not yet fully elucidated^(1)^. Recent studies have shown that CXL treatment can improve corneal rigidity^(2,3)^ and increase corneal resistance to enzymatic digestion^(4)^.

Conventional CXL, described as the “Dresden protocol,” includes epithelial debridement followed by corneal saturation with riboflavin solution. Following these steps, the cornea is exposed to UVA radiation (370 nm) at 3 mW/cm^2^ for 30 min, thereby achieving a surface dose of 5.4 J/cm^2^(^5)^. Conventional CXL treatment is preferred in eyes with corneal thicknesses of ≥400 µm after epithelial debridement, in order to prevent endothelial toxicity^(6,7)^. However, most keratoconic corneas that require CXL may have corneal thicknesses of <400 µm. Prolonged UVA exposure may lead to corneal dehydration during CXL, accompanied by reduced corneal thickness^(8)^. Hence, various modifications of the CXL procedure have been developed to avoid complications during the CXL of thin corneas. These modifications include CXL with hypo-osmolar riboflavin, transepithelial CXL, iontophoresis-assisted CXL, CXL with customized epithelial debridement, lenticule-assisted CXL, contact lens-assisted CXL, and individualized CXL.

In addition to modifications regarding the CXL procedure, various formulations of riboflavin solutions have been shown to play important roles in the potential efficacy of CXL treatment. In conventional CXL, iso-osmolar riboflavin solutions are effective; the de-epithelialized cornea must be ≥400 µm for these solutions to be used. Hence, techniques to induce the swelling of thin corneas have been investigated. Maurice and Giardini showed that corneal irrigation with hypo-osmolar solutions can cause swelling to double the normal thickness^(9)^. Preoperative swelling of thin corneas improves the outcomes of CXL in patients who would otherwise not be eligible for this type of treatment.

The conventional CXL protocol (3 mW/cm^2^ for 30 min) is effective in slowing down the progression of keratoconus; an accelerated CXL protocol (9 mW/cm^2^ for 10 min) has also been shown to yield similar outcomes. Reducing the duration of the procedure could relieve patient discomfort and reduce the occurrence of adverse effects related to prolonged corneal exposure^(10,11)^. In this study, we investigated the effectiveness of an accelerated CXL protocol using hypo-osmolar riboflavin solution and UVA for the treatment of keratoconus in patients with a de-epithelialized corneal thickness of <400 µm.

## METHODS

### Patients and clinical assessments

This retrospective nonrandomized study was approved by the local Ethics Committee in our institution. Informed consent was obtained from each patient. This study included 28 eyes of 24 patients who had corneal stromal thicknesses between 330 and 400 µm, had progressive keratoconus, and had undergone epithelium-off accelerated CXL. Progression criteria were defined as progression >1 D (diopter) in maximum keratometry (Kmax), increased thinning of >2% on the thinnest section of the cornea, and a >0.5 D increase in manifested spherical refractive values on a Scheimpflug camera (Pentacam HR, Oculus Optik geräte GmbH, Wetzlar, Germany) evaluation at 12 months post-CXL. Patients were excluded if they had any history of previous anterior segment surgery, ocular surface problems, or herpetic keratitis; patients were also excluded if they had active ophthalmic infection or any central/paracentral corneal scarring.

The following patient data were recorded before CXL and at 1, 6, 12, and 24 months postoperatively: medical history, uncorrected visual acuity (UCVA), best-corrected visual acuity (BCVA) (with eyeglasses), letter contrast sensitivity (CS), slit-lamp and fundus examination findings, Kmax, total corneal astigmatism, flattest and steepest keratometric (K) readings, central corneal thickness at the thinnest point (t-CCT), and corneal higher-order aberration (HOA) values from corneal topographic analysis. Examination quality was evaluated in each topographical image; images were used if the quality was “ok.”

Visual acuity was recorded in logMAR, using the Smart System 2 2020 Visual Acuity System (M&S Technologies). CS was evaluated by the Pelli-Robson chart, which includes horizontal lines of capital letters organized into groups of three (triplets) with two triplets per line. The contrast decreases sequentially among triplets, even within each line. All patients were assessed under monocular vision at a distance of 1 m from the chart and under controlled photopic conditions (85 cd/m^2^). Patients were asked to read the letters, and the values were recorded as log CS [log (l/c)]. Corneal HOAs were measured over a 6.0-mm-diameter central corneal zone. The Pentacam system software was used to calculate aberrations of the anterior corneal surface using Zernike polynomials with expansion up to the sixth order. The root-mean-square (RMS) values of HOAs were expressed in micrometers.

### Surgical procedure and postoperative treatment

Before the CXL operation, proparacaine hydrochloride (0.5%) (Alcaine, Alcon Laboratories, Puurs, Belgium) was applied. The operation was initiated with debridement of the central 8.0 mm of corneal epithelium by a crescent knife (Beaver-Visitec International Inc., Waltham, MA) with the assistance of 20% alcohol application for 30 s. Subsequently, 0.9% NaCl solution was used for a thorough cleaning of the corneal surface. Hypo-osmolar 0.1% riboflavin (0.25% riboflavin, 1.2% HPMC, 0.01% benzalkonium chloride, MedioCROSS^®^ H, Avedro Inc., USA) was applied at 2-min intervals immediately after removal of the epithelium until the minimal corneal thickness reached 400 mm.

Corneal pachymetry was measured using an ultrasound probe (SP-2000, Tomey, Inc.) before CXL, after epi thelial removal, and every 10 min thereafter. Ten mea surements were recorded in the area of thinnest corneal thickness at each time point; the lowest pachymetry value was recorded during each measurement. Blue-light slit-lamp biomicroscopy was used to ensure successful penetration of riboflavin through the cornea by visualizing it in the anterior chamber. Riboflavin was continuously applied at 2-min intervals during the course of a 10-min exposure to 9 mW/cm^2^ UVA (Cross-K, NIDEK, Italy). Finally, a therapeutic contact lens (Air Optics, Alcon, Inc.) was fitted. Postoperatively, both eyes were treated with diclofenac (Acular LS^®^) 4× per day, netilmicin (Netira^®^) 4× per day, Loteprednol (Lotemax^®^, Bausch and Lomb Inc.) 6× per day, and artificial tears 6× per day. Patients were followed up on a daily basis until complete re-epithelialization was observed. Topical netilmicin was used for 1 week, loteprednol was used with a tapering schedule for 3 months, and artificial tears were continued for 6 months.

### Statistical analysis

Data were analyzed using SPSS 20.0 for Mac (SPSS, Inc., Chicago, IL). Mean ± SD was used for descriptive statistics. Paired sample t-tests were performed to compare preoperative and postoperative astigmatism, t-CCT, UCVA, BCVA, CS, and DCS. A p value of <0.05 was considered to be statistically significant.

## RESULTS

Twenty-eight eyes of 24 patients (13 women, 11 men) underwent epithelium-off accelerated CXL. The mean age of the patients was 25.3 ± 5.1 (range, 19-35) years. The clinical features of the patients are shown in table 1.

**Table 1:** Clinical features of patients, preand post-CXL

N=28	Pre-CXL	1 month post-CXL	6 months post-CXL	12 months post-CXL	24 months post-CXL
Astigmatism (D) ± SD	4.82 ± 1.47	4.93 ± 1.34	4.71 ± 0.16	4.56 ± 0.19^*^	4.53 ± 0.11
Flattest K (D) ± SD	47.41 ± 2.45	48.23 ± 0.45	47.45 ± 0.36	47.21 ± 1.03	47.25 ± 2.9
Steepest K (D) ± SD	59.12 ± 14.12	58.45 ± 11.78	58.56 ± 0.23^*^	57.23 ± 4.25^*^	57.03 ± 3.04^*^
t-CCT ± SD	410.5 ± 11.6	400.3 ± 6.56	405.36 ± 45.41	403.21 ± 22.5	403.16 ± 18.17
UCVA ± SD	1.02 ± 0.15	1.34 ± 0.78	0.98 ± 0.34	0.91 ± 0.10	0.92 ± 0.08
BCVA ± SD	0.71 ± 0.1	0.81 ± 1.45	0.65 ± 0.56^*^	0.52 ± 0.12^*^	0.51 ± 0.46^*^
CS ± SD	0.83 ± 0.01	0.85 ± 0.04	0.86 ± 0.05	0.97 ± 0.05^*^	0.98 ± 0.01^*^

* Statistically significant, pre-CXL versus post-CXL.

Values are given as mean ± standard deviation (SD).

UCVA= uncorrected visual acuity (logMAR); BCVA= best-corrected visual acuity (logMAR); t-CCT= thinnest point of central corneal thickness (µm); CS= contrast sensitivity (logMAR).

Although there was no change in the mean flattest K measurement, the mean steepest K values showed significant reductions at 6, 12, and 24 months postoperatively. These reductions led to improved BCVAs at 6, 12, and 24 months postoperatively (p=0.013, p=0.025, and p=0.021, respectively). In addition, the reduction in the steepest K values resulted in reductions of total astigmatism at 24 months postoperatively (p=0.002 and p=0.035). These changes were not accompanied by any reductions in t-CCT values, which indicates that the method was reliable (p=0.12). The respective mean CSs in logMAR were 0.97 ± 0.05 and 0.98 ± 0.01 at 12 and 24 months postoperatively; these values were significantly higher than the pre-CXL values (p=0.038 and p=0.033, respectively). During treatment, t-CCT was reduced to 364.21 ± 22.5 µm after epithelium removal; it was increa sed to 415.34 ± 12.45 µm (range, 400-430 µm) using hypotonic solution.

Table 2 shows the HOA values of patients before CXL and at 1, 6, 12, and 24 months postoperatively. Compared with the values before CXL, significant reductions were detected in vertical coma and spheric aberration (SA) at 6, 12, and 24 months postoperatively (vertical coma: p=0.042, p=0.023, and p=0.021, respectively; SA: p=0.032, p=0.027, and p=0.016, respectively); moreover, the horizontal and total RMS values were significantly reduced at 12 and 24 months postoperatively (horizontal RMS: p=0.024 and p=0.025, respectively; total RMS: p=0.041 and p=0.037, respectively).

**Table 2:** Higher-order aberrations, preand post-CXL

N=28	Pre-CXL	1 month post-CXL	6 months post-CXL	12 months post-CXL	24 months post-CXL
SA (µm) ± SD	-0.98 ± 0.12	-1.08 ± 0.25	-0.81 ± 0.16^*^	-0.77 ± 0.18^*^	-0.77 ± 0.26^*^
Vertical coma (µm) ± SD	-2.01 ± 0.14	-2.11 ± 0.23	-1.89 ± 0.13^*^	-1.63 ± 0.25^*^	-1.62 ± 0.16^*^
Horizontal coma (µm) ± SD	-0.40 ± 0.18	-0.39 ± 0.04	-0.37 ± 0.03	-0.33 ± 0.23^*^	-0.33 ± 0.04^*^
Total RMS (µm) ± SD	-4.38 ± 1.12	-4.22 ± 0.10	-4.04 ± 0.14	-3.82 ± 0.19^*^	-3.80 ± 0.28^*^

* Statistically significant, pre-CXL versus post-CXL.

Values are shown as mean ± standard deviation (SD). SA= spheric aberration.

Therapeutic contact lenses were well tolerated by all patients. No problems regarding epithelial healing were observed after treatment; in addition, no complications were noted, such as infection, contact lens damage, or corneal infiltration.

## DISCUSSION

Corneal ectasia is an irregular protrusion of the cornea that results from changes in the stromal collagen matrix. Primary forms of corneal ectasia are keratoconus, pellucid marginal degeneration, and keratoglobus; secondary forms include ectasia after refractive surgery^(1)^. Depending on the activity and stage of keratoconus, the approaches utilized to improve visual acuity include eyeglasses, contact lenses, intrastromal corneal ring seg ments, phakic intraocular lenses, photorefractive ker atectomy, and corneal transplantation^(12)^. In general, keratoconus progression is controlled by improving the biomechanical strength and stability of the cornea with CXL^(13)^. The application of riboflavin and UVA to reform new covalent bands is usually performed in the relatively new approach of CXL. At various stages of keratoconus, the rates of CXL complications vary from 1% to 10%. Transient stromal haze, sterile infiltrates, endothelium decompensation, delayed epithelial healing, and infectious keratitis are among the early postoperative complications. Late postoperative complications can include stromal opacity^(14)^.

Endothelium decompensation is an important complication of CXL. When the cornea is saturated with riboflavin to a thickness of 400 µm, UVA radiance at the endothelial level is below the cytotoxic level. This suggests that a de-epithelialized corneal stromal thickness of 400 µm should be used as a safety limit to protect the endothelium and intraocular structures from the adverse effects of UVA irradiation. Therefore, a number of different methods have been proposed to protect against toxicity in patients with corneal thicknesses <400 µm. New techniques include the use of hypo-osmolar riboflavin solution to cause corneal swelling during the operation, transepithelial CXL without the removal of corneal epithelium, and customized epithelial debridement^(15,16)^. Under physiological conditions, a swelling pressure of 50-60 mm Hg is observed in the corneal stroma. Stromal swelling to double thickness can occur as a result of the irrigation of the corneal stroma with a solution that has lower colloid osmotic pressure (hypo-osmolar solution)^(17)^. During the first stage of the CXL procedure, irrigation of the corneal stroma is used to increase the corneal thickness, following de-epithelialization^(9)^. In the current study, the thickness of the de-epithelialized cornea increased from 364.21 ± 22.5 to 410.34 ± 12.45 µm when using hypo-osmolar riboflavin solution.

In the present study, we aimed to examine the outcomes of accelerated CXL using hypo-osmolar riboflavin solution in thin corneas for a follow-up period of 24 months. We used hypo-osmolar dextran-free riboflavin combined with 9 mW/cm^2^ UVA; we evaluated changes in corneal parameters and visual performance. The use of hypotonic riboflavin solution increased corneal pachymetry, although the final t-CCT remained unchanged. In addition, our results are compatible with those reported in previous studies using hypotonic riboflavin solution^(18)^. In both our study and the previous study, there were no statistically significant changes in the t-CCT values measured before and after CXL. As reported in previous studies^(19,20)^, the use of hypo-osmolar riboflavin solution in the current study did not impair the efficiency of treatment in terms of corneal parameters and visual performance. The hydrophilic capacity of proteoglycans between collagen fibers leads to stromal swelling, which increases the distance between collagen fibrils. This increased distance prevents the formation of covalent bonds between collagen fibrils.

No keratometry complications due to the utilization of hypo-osmolar riboflavin solution have been reported previously in the literature; moreover, keratometry sta bilization has been described in some previous studies^(19-25)^. In a study of accelerated corneal collagen cross-linking (30 mW/cm^2^ at 3 min) in patients with corneal thicknesses <400 µm, Ozgurhan et al. reported that the flattest keratometry readings decreased from 47.40 ± 2.52 to 46.95 ± 2.48 D, whereas the steepest keratometry readings decreased from 51.04 ± 3.71 to 50.62 ± 3.57 D^(26)^. In the current study, the flattest keratometry readings decreased from 47.41 ± 2.45 to 47.25 ± 2.9 D, whereas the steepest keratometry readings decreased from 59.12 ± 14.12 to 57.03 ± 3.04 D at the end of the 24-month follow-up period. Some clinical trials have reported that CXL may cause limited regression of corneal protrusion^(27-32)^. Flattening of the cornea may occur after standard and accelerated CXL, potentially at ≥1 year postoperatively^(33,34)^. Flattening of the protruded cornea may be more remarkable in patients with advanced cases of keratoconus. Chen et al. reported that higher preoperative keratometry values were associated with greater corneal flattening after CXL^(35)^. In the present study, statistically significant reductions in the steepest K readings occurred at 6, 12, and 24 months after CXL, and no changes were observed in the flattest keratometry readings.

No complications were observed in relation to the use of hypo-osmolar riboflavin solution. Previously, Ozgurhan et al. found that UCVA improved from 0.67 ± 0.32 logMAR to 0.56 ± 0.28 logMAR and BCVA improved from 0.49 ± 0.19 logMAR to 0.42 ± 0.19 logMAR, although these improvements were not statistically significant at 12 months postoperatively. In another study that used hypotonic riboflavin in thin corneas, the mean BCVA of 0.54 ± 0.23 logMAR improved to 0.52 ± 0.1 logMAR (p=1) at 6 months postoperatively and to 0.51 ± 0.21 logMAR (p=0.285) at 12 months postoperati vely^(26)^. The mean BCVAs showed no significant changes at these follow-up visits, compared with the values recorded before CXL (all p>0.05)^(36)^. Koç et al. evaluated 49 eyes of 43 patients who had progressive keratoconic thin corneas and underwent accelerated corneal CXL with hypo-osmolar riboflavin; they also concluded that accelerated corneal CXL with hypo-osmolar riboflavin solution was effective in thin corneas^(37)^.

In the current study, UCVA increased from 1.02 ± 0.15 logMAR to 0.98 ± 0.34 logMAR at 6 months postoperatively, but this change was not statistically significant. However, a statistically significant increase was observed in BCVA during the 24-month follow-up period (from 0.71 ± 0.1 logMAR to 0.51 ± 0.46 logMAR), which could be attributed to the longer period of examination.

CXL was found to be effective in improving visual acuity and CS; corneal aberrations were also reduced. This indicates that CXL can improve visual quality and visual acuity. Notably, these results emphasize that hy potonic riboflavin solution is effective in the CXL treat ment of thin corneas. Especially in patients with thin corneas who have advanced cases of keratoconus, CXL treatment can stop the progression of disease; thus, fewer patients may be referred for keratoplasty, which is a more invasive and complicated treatment procedure.
